# Yes-Associated Protein (YAP) Promotes Tumorigenesis in Melanoma Cells Through Stimulation of Low-Density Lipoprotein Receptor-Related Protein 1 (LRP1)

**DOI:** 10.1038/s41598-017-14764-4

**Published:** 2017-11-14

**Authors:** Huizi Xiong, Qian Yu, Yu Gong, Wenjuan Chen, Yunlei Tong, Yao Wang, Hui Xu, Yuling Shi

**Affiliations:** 1 Department of Dermatology, Shanghai Tenth People’s Hospital, Tongji University School of Medicine, Shanghai, 200072 China; 2 Department of Dermatology, The Affiliated Hospital of Jiangnan University, Wuxi, Jiangsu 214062 China

## Abstract

YAP is a critical protein in cancer development and can induce transformative phenotypes in mammary epithelial cells. Previous studies have provided evidence that YAP can contribute to the metastatic behavior of melanoma, since specific knockdown of YAP leads to reduced metastatic and invasive capacity *in vitro*. However, the mechanism by which YAP regulates the function of melanoma is unknown. Here, we identified that YAP has a positive impact on the expression of LRP1, which also plays critical roles in cancer. Mechanically, knockdown of YAP resulted in a significant down-regulation of LRP1 at both the protein and mRNA levels. Tissue microarray analysis (TMA) also showed a positive correlation between YAP and LRP1 expression. In addition, reduction of YAP-impaired pro-carcinogenic phenotypes could be partially reversed by simultaneous overexpression of LRP1, suggesting that LRP1 is functionally important in YAP-induced melanoma tumorigenesis. Furthermore, we found that LRP1 was regulated by YAP through a transcription- and promoter-dependent mechanism. Taken together, our results suggest that YAP regulates LRP1 through stimulation of the LRP1 promoter and that LRP1 may be an important target for influencing YAP in melanoma.

## Introduction

Cutaneous melanoma is one of the most severe skin cancers and occurs when cells in the skin develop abnormally. The incidence of melanoma is increasing in many developed countries, since this form of cancer occurs predominantly in pale-skinned people who expose themselves to intense sunlight, especially when vacationing in sunny places^1,2^. According to Cancer Research UK, approximately 2,100 people died from malignant melanoma in 2012, and this disease accounted for more deaths from cancer than all the other skin cancer types combined. In 2011, 13,300 cases of malignant melanoma were diagnosed, with rates rising more than fivefold since the mid-1970s. There is an urgent demand for the development of more effective treatment strategies as well as the elucidation of the associated molecular mechanisms.

Melanoma is an uncommon cutaneous carcinoma, but it has a high risk of metastasis and a high mortality rate. Malignant melanoma is a highly aggressive form of skin cancer, the incidence of which is rising rapidly^3^. The incidence of cutaneous melanoma varies around the world. In western countries, cutaneous melanoma is a relatively common malignancy, especially in populations with lighter skin colors. According to the International Agency for Research on Cancer, the incidence of cutaneous melanoma is highest in Queensland, Australia, but it is also high in Auckland, New Zealand^4^. In the USA, cutaneous melanoma is the fifth most commonly diagnosed cancer^5^. The incidence of cutaneous melanoma is low in Asian populations, but there is a definite increasing trend in Asians. Malignant melanoma can occur in any melanocyte-containing anatomic site. Four main cutaneous melanoma subtypes are recognized: lentigo maligna melanoma, nodular melanoma, superficial spreading melanoma, and acral lentiginous melanoma (ALM). In Asians, ALM is the most common type of melanoma, but it is usually detected during later stages than other melanoma types. Generally, excessive exposure to ultraviolet (UV) radiation increases the risk of developing melanoma. The exception to this is ALM, which is the most common melanoma subtype in Asians and is not associated with UV radiation^6^.

YAP has the biological function of mediating gene regulation in the tumor-suppressing Hippo signaling pathway. YAP is amplified in many human cancers and promotes cell proliferation and cell morphologic transition. Previous studies have provided evidence that YAP contributes to maintaining the transformative phenotypes of melanoma cells^3,4^. Furthermore, it has been revealed that YAP plays an essential role in Gq/11-induced tumorigenesis and that YAP can be treated as a potential drug target for uveal melanoma (UM) accompanied by mutations in GNAQ or GNA11^7^. However, the mechanism underlying how YAP promotes initiation and progression of melanoma remains unknown.

LRP1 is a member of the low-density lipoprotein receptor (LDLR) family involved in the metabolism of various extracellular ligands, including proteinases and lipoproteins that play a vital role in melanoma tumorigenesis. Some studies have strongly suggested that LRP1 promotes glioblastoma cell migration and invasion by regulating the expression and function of MMP2 and MMP9 through an ERK-dependent signaling pathway^8^. Moreover, LRP1 has important roles in the regulation of cell growth, cell migration, and tissue remodeling through the regulation of extracellular proteolytic activity^9^. However, whether and how LRP1 and YAP are closely associated with each other in melanoma cells is poorly understood.

As we know, cutaneous malignant melanoma is the most fatal skin cancer, and although improved understanding of its pathogenic pathways has allowed us to develop some effective molecular-targeted therapies, novel targets and drugs are still needed. In the present study, we have identified a significant new target, LRP1, which may be important in YAP-induced melanoma tumorigenesis.

## Results

### Both YAP and LRP1 promote transformative phenotypes in melanoma A375 and MUM-2B cells

**Figure 1:**
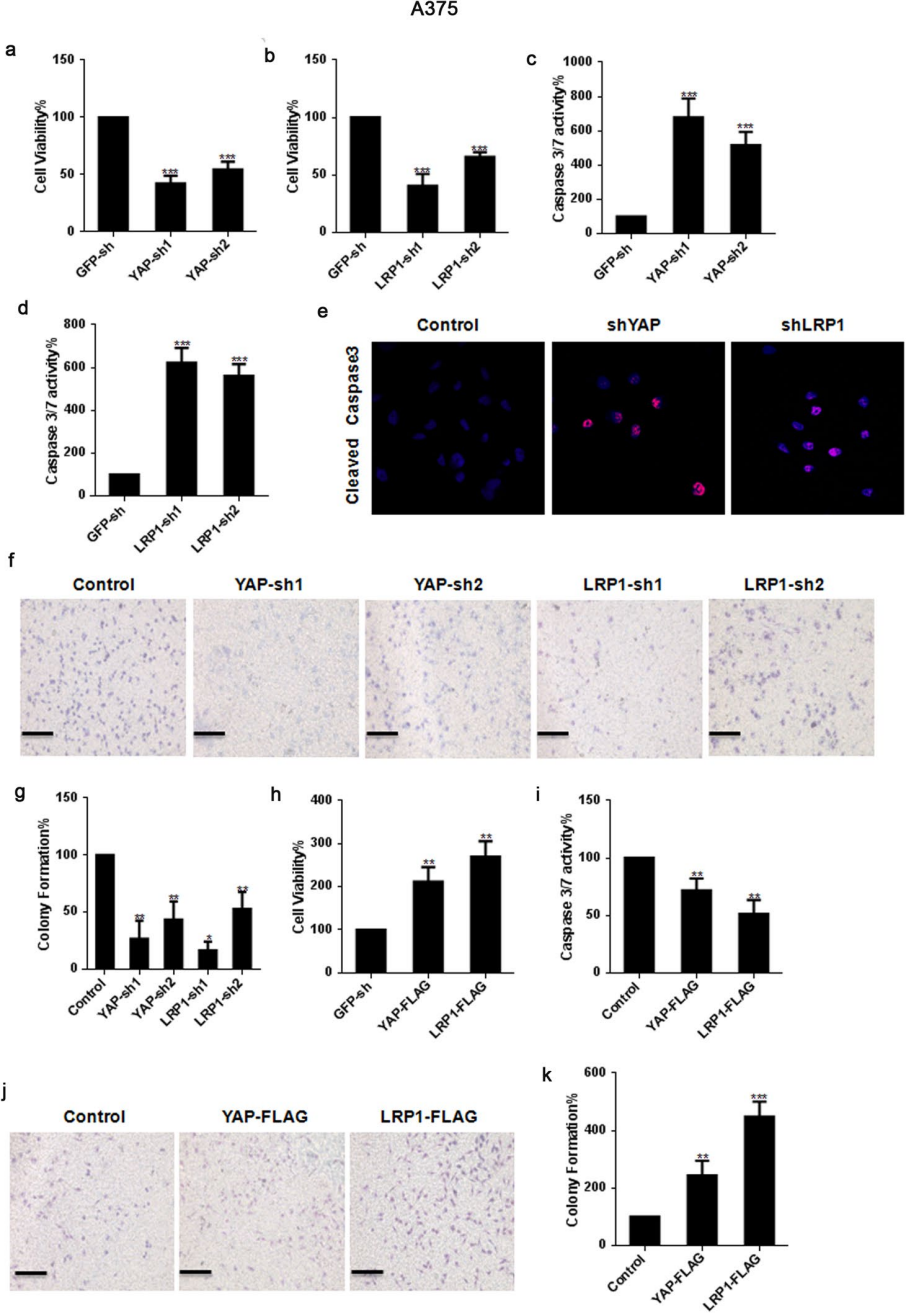
YAP and LRP1’s function in promoting transformative phenotypes in melanoma A375 cells. (**a**,**b**) Cell proliferation of melanoma A375 cells under infection of YAP-sh plasmid or LRP1-sh plasmid as indicated were evaluated using MTT assay. The initial cell number is 5000 for MTT assay, and the data from the “GFP-sh” group were arbitrarily set to 100%. (**c**,**d**) Caspase 3/7 activities of melanoma cells under infection of YAP-sh plasmid or LRP1-sh plasmid as indicated were measured by a Caspase-Glo 3/7 assay kit from Promega. (**e**) YAP and LRP1 reduced Caspase 3 expression, as measured by immunofluorescence assay, in A375 cells infected of YAP-sh plasmid or LRP1-sh plasmid for 24 h. Scale bar.μm. (**f**,**g**) Cell proliferation of melanoma A375 cells under infection of YAP-sh plasmid or LRP1-sh plasmid as indicated were evaluated using transwell assay. The initial cell number is 5000 for transwell assay, and the data from the “GFP-sh” group were arbitrarily set to 100%. (**h**) Cell proliferation of melanoma A375 cells under transfection of YAP-FLAG plasmid or LRP1-FLAG plasmid as indicated were evaluated using MTT assay respectively. (**i**) Caspase 3/7 activities of melanoma cells under transfection of YAP-FLAG plasmid or LRP1-FLAG plasmid as indicated were measured by a Caspase-Glo 3/7 assay kit from Promega. (**j**,**k**) Cell proliferation of melanoma A375 cells under transfection of YAP-FLAG plasmid or LRP1-FLAG plasmid as indicated were evaluated using MTT assay. Data were shown as mean ± SD from three independent experiments (including WB). *P < 0.05; **P < 0.01; ***P < 0.001 versus control measured by the student *t* test.

**Figure 2:**
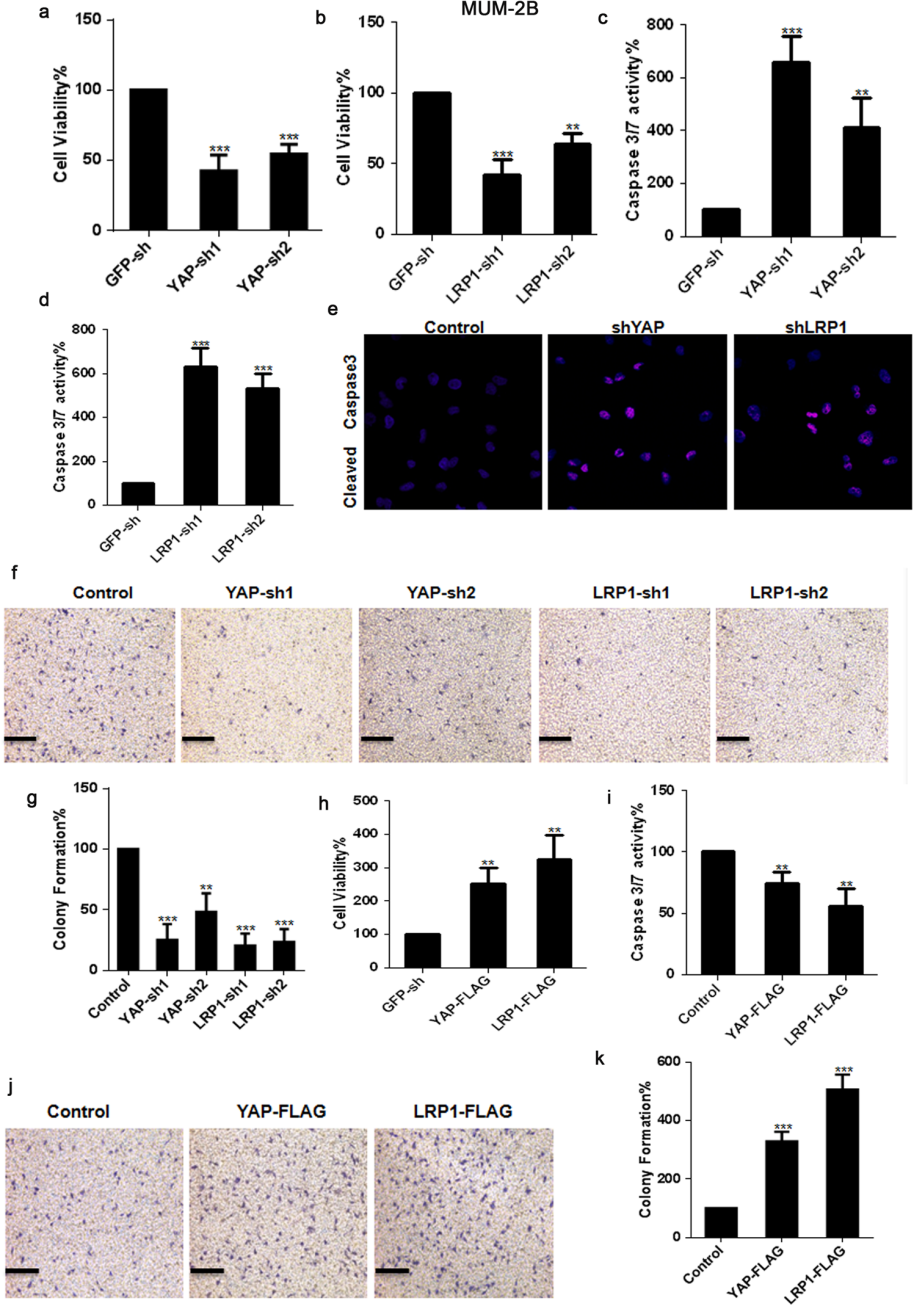
YAP and LRP1’s function in promoting transformative phenotypes in melanoma MUM-2B cells. (**a**,**b**) Cell proliferation of melanoma MUM-2B cells under infection of YAP-sh plasmid or LRP1-sh plasmid as indicated were evaluated using MTT assay respectively. The initial cell number is 5000 for MTT assay, and the data from the “GFP-sh” group were arbitrarily set to 100%. (**c**,**d**) Caspase 3/7 activities of melanoma cells under infection of YAP-sh plasmid or LRP1-sh plasmid as indicated were measured by a Caspase-Glo 3/7 assay kit from Promega. (**e**) YAP and LRP1 reduced Caspase 3 expression, as measured by immunofluorescence assay, in MUM-2B cells infected of YAP-sh plasmid or LRP1-sh plasmid for 24 h. Scale bar.μm. (**f**,**g**) Cell proliferation of melanoma MUM-2B cells under infection of YAP-sh plasmid or LRP1-sh plasmid as indicated were evaluated using transwell assay. The initial cell number is 5000 for transwell assay, and the data from the “GFP-sh” group were arbitrarily set to 100%. (**h**) Cell proliferation of melanoma MUM-2B cells under transfection of YAP-FLAG plasmid or LRP1-FLAG plasmid as indicated were evaluated using MTT assay. (**i**) Caspase 3/7 activities of melanoma cells under transfection of YAP-FLAG plasmid or LRP1-FLAG plasmid as indicated were measured by a Caspase-Glo 3/7 assay kit from Promega. (**j**,**k**) Cell proliferation of melanoma MUM-2B cells under transfection of YAP-FLAG plasmid or LRP1-FLAG plasmid as indicated were evaluated using transwell assay. Data were shown as mean ± SD from three independent experiments. *P < 0.05; **P < 0.01; ***P < 0.001 versus control measured by the student *t* test.

To investigate the relationship between YAP and LRP1, we first knocked down YAP and LRP1 in the A375 cells and MUM-2B cells. We found that knockdown of YAP resulted in an inhibition of cell proliferation in A375 cells and MUM-2B cells, as measured by an MTT-based assay (Figs 1a and 2a). Interestingly, a similarly impaired cell proliferation was also detected when LRP1 was knocked down (Figs 1b and 2b). Moreover, knockdown of YAP increased caspase-3/7 activity compared to the control (Figs 1c and 2c). As expected, knockdown of LRP1 also led to an increase in Caspase-3/7 activity (Figs 1d,e and 2d,e). In addition, we found both knockdown of YAP and knockdown of LRP1 led to reduced cell migration compared to the control, as determined by a transwell assay (Figs 1f,g and 2f,g). In contrast, overexpression of either YAP or LRP1 induced cell proliferation (Figs 1h and 2h), reduced caspase-3/7 activity (Figs 1i and 2i) and induced transwell activity (Figs 1j,k and 2j,k). These data suggested that YAP and LRP1 might closely cooperate and play similar roles in promoting transformative phenotypes in melanoma A375 cells and MUM-2B cells.

### Both YAP and LRP1 levels were elevated and were closely associated in melanoma

**Figure 3:**
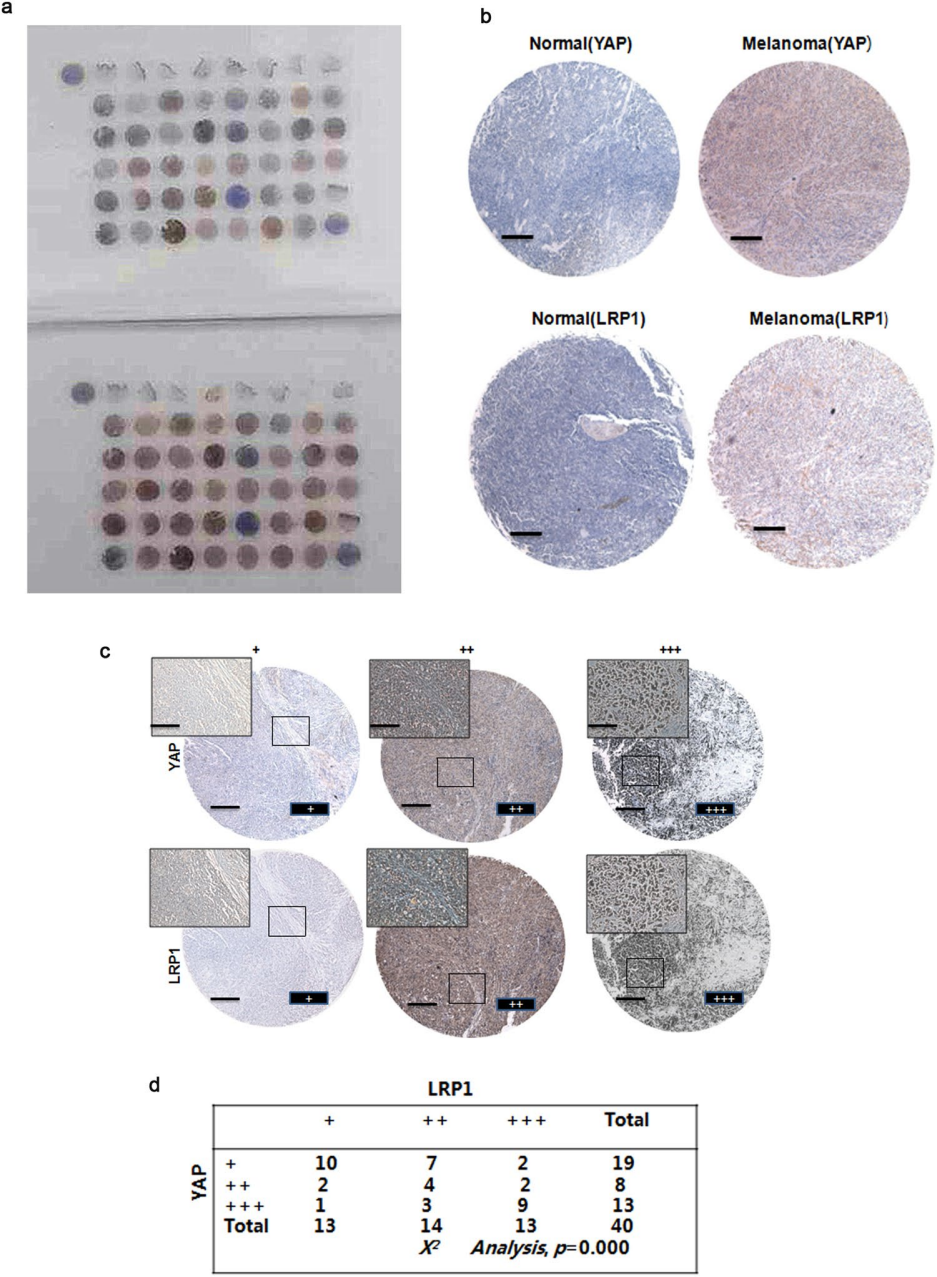
The consistency of YAP and LRP1 in tissue microarray specimen. (**a**,**b**) TMA slides include forty skin melanoma tissues and eight skin normal tissues which locate on the bottom of the each TMA. Representative images of IHC from HCC TMA stained with anti-YAP or anti-LRP1 antibodies. Scale bar, 100 μM. (**c**) Representative images of IHC from skin melanoma HCC TMA stained with anti-YAP or anti-LRP1 antibodies. Scale bar, 100 μM. (**d**) The statistical figure of skin melanoma IHC images from HCC TMA stained with anti-YAP or anti-LRP1 antibodies. The TMA data were analyzed using the χ2 test.

In the previous experiments, we revealed that YAP and LRP1 play similar roles in maintaining transformative phenotypes in melanoma A375 cells and MUM-2B cells. However, the relationship between YAP and LRP1 in clinical specimens had not been confirmed. By testing a series of melanoma and normal skin tissues on TMA slides using IHC, we found that both YAP and LRP1 levels were highly elevated in melanoma tissues compared to normal skin tissues (Fig. 3a). Interestingly, higher expression levels of YAP were correlated with higher expression levels of LRP1 in melanoma tissues (Fig. 3b,c), suggesting the importance of the collaboration between YAP and LRP1 in clinical melanoma samples.

### YAP-promoted LRP1 was dependent on transcription in the A375 cells and MUM-2B cells

**Figure 4:**
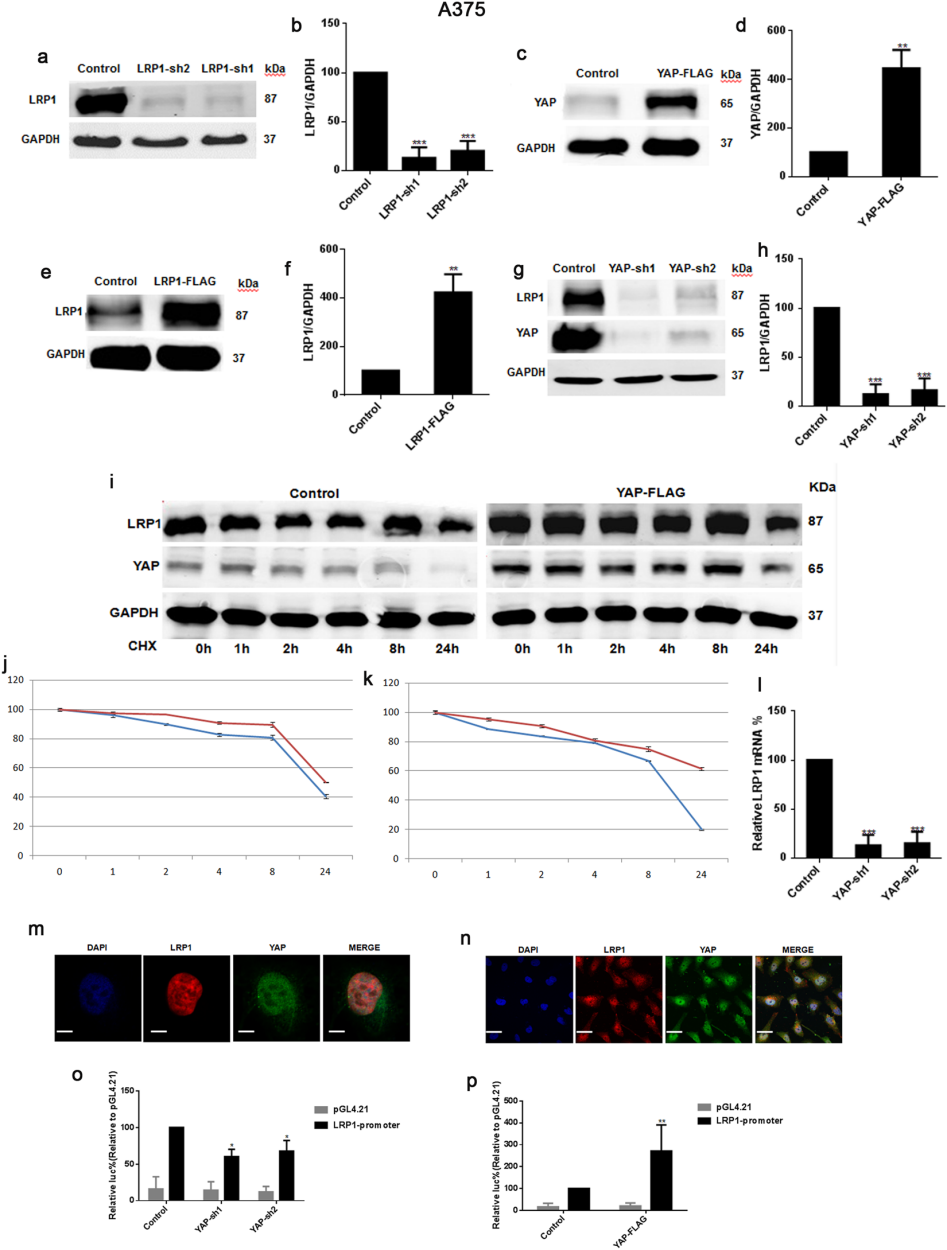
YAP -promoted LRP1 was depended on transcription in the A375 cells. (**a**,**b**) Western blots of LRP1 in melanoma A375 cells infected with GFP-sh or LRP1-sh1or LRP1-sh2 (**a**); relative LRP1 protein levels were shown as the ratio between LRP1 and GAPDH, and protein levels of the A375 cells infected with GFP-sh was arbitrarily set to 100% (**b**). (**c**,**d**) Western blots of YAP in melanoma A375 cells a transfected with GFP-sh or YAP-FLAG (**c**); relative LRP1 protein levels were shown as the ratio between YAP and GAPDH, and protein levels of melanoma A375 cells infected with GFP-sh were arbitrarily set to 100% (**d**). (**e**,**f**) Western blots of LRP1 in melanoma A375 cells transfected with GFP-sh or LRP1-FLAG (**e**); relative LRP1 protein levels were shown as the ratio between LRP1 and GAPDH, and protein levels of the A375 cells infected with GFP-sh were arbitrarily set to 100% (**f**). (**g**,**h**) Western blots of LRP1 in melanoma A375 cells infected with GFP-sh or YAP-sh1or YAP-sh2 (**g**); relative LRP1 protein levels were shown as the ratio between LRP1 and GAPDH, and protein levels of the A375 cells infected with GFP-sh were arbitrarily set to 100% (**h**). (**i**) The LRP1 protein in melanoma A375 cells transfected with empty or YAP-FLAG plasmids was detected using Western blot after adding CHX (50 μg/ml) for indicated time (upper panel). And the LRP1 protein was normalized to that of GAPDH. The 0 h time point was arbitrarily set to 100% (lower panel). (**j**) Time-dependent Caspase 3/7 activities after CHX (50 μg/ml) of LRP1 in the indicated A375 cells. The blue line was represented the control group; The red line was represented the YAP-FLAG group. (**k**) Time-dependent Caspase 3/7 activities after CHX (50 μg/ml) of YAP in the indicated A375 cells. The blue line was represented the control group; The red line was represented the YAP-FLAG group. (**l**) Relative mRNA of LRP1 in melanoma A375 cells infected with GFP-sh or YAP-sh1 or YAP-sh2.(**m**,**n**) Immunofluorescence assays using anti-YAP and anti-LRP1 antibodies (lower panel). Scale bar, 10 μM. (**o**,**p**) Knockdown of YAP inhibited the activity of LRP1 promoter and over-expressed YAP stimulated the activity of LRP1 promoter, LRP1 promoter containing −2000 bp to +150 bp luciferase activities were measured in melanoma A375 cells with control which were transfecting PGL4/Basic. Data were shown as mean ± SD from three independent experiments (including WB). *P < 0.05; **P < 0.01; ***P < 0.001 versus control measured by the student *t* test.

**Figure 5:**
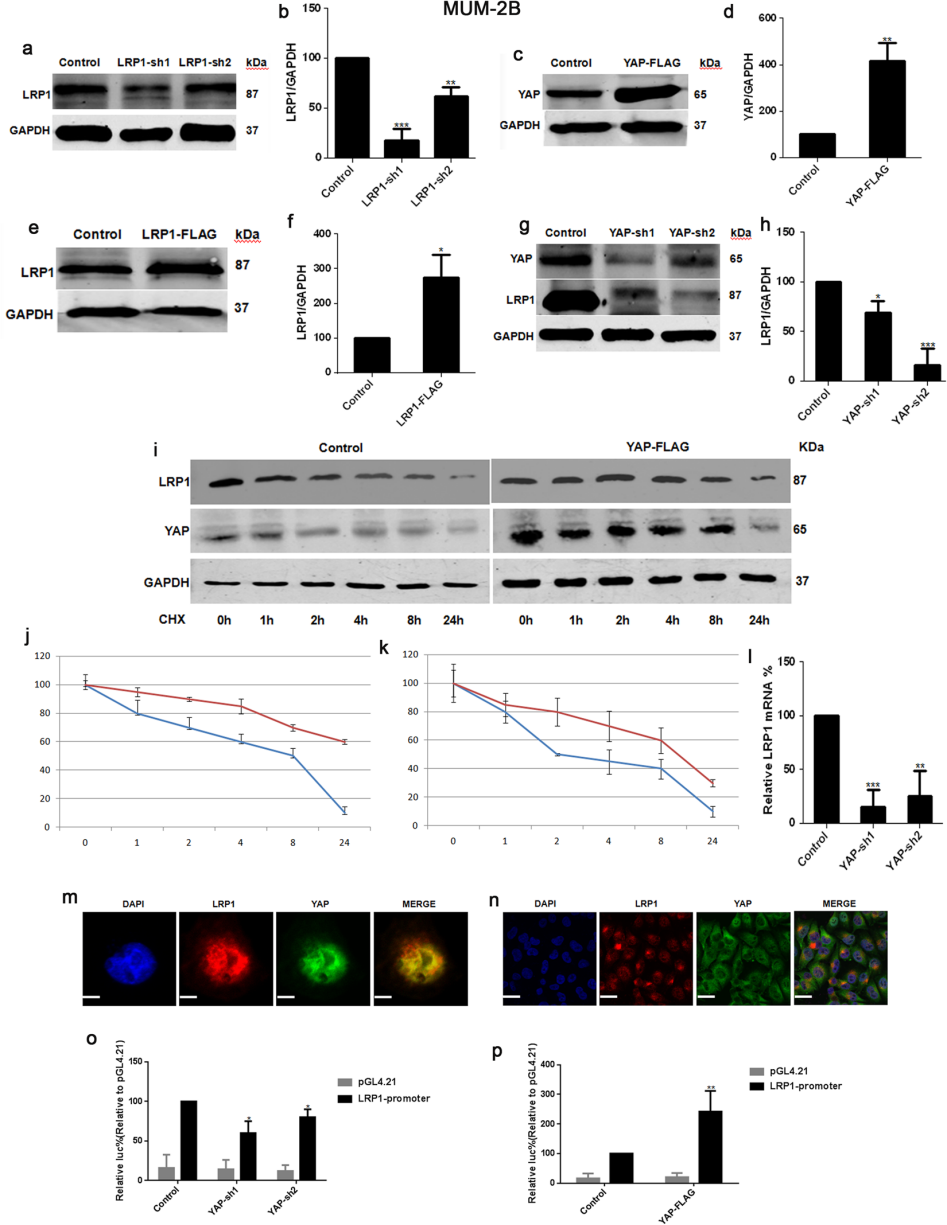
YAP -promoted LRP1 was depended on transcription in the MUM-2B cells. (**a**,**b**) Western blots of LRP1 in melanoma MUM-2B cells infected with GFP-sh or LRP1-sh1or LRP1-sh2 (**a**); relative LRP1 protein levels were shown as the ratio between LRP1 and GAPDH, and protein levels of the MUM-2B cells infected with GFP-sh was arbitrarily set to 100% (**b**). (**c**,**d**) Western blots of YAP in melanoma MUM-2B cells transfected with GFP-sh or YAP-FLAG (**c**); relative LRP1 protein levels were shown as the ratio between YAP and GAPDH, and protein levels of melanoma MUM-2B cells infected with GFP-sh were arbitrarily set to 100% (**d**). (**e**,**f**) Western blots of LRP1 in melanoma MUM-2B cells transfected with GFP-sh or LRP1-FLAG (**e**); relative LRP1 protein levels were shown as the ratio between LRP1 and GAPDH, and protein levels of the MUM-2B cells infected with GFP-sh were arbitrarily set to 100% (**f**). (**g**,**h**) Western blots of LRP1 in melanoma MUM-2B cells infected with GFP-sh or YAP-sh1or YAP-sh2 (**g**); relative LRP1 protein levels were shown as the ratio between LRP1 and GAPDH, and protein levels of the MUM-2B cells infected with GFP-sh were arbitrarily set to 100% (**h**). (**i**)The LRP1 protein in melanoma MUM-2B cells transfected with empty or YAP-FLAG plasmids was detected using Western blot after adding CHX (50 μg/ml) for indicated time (upper panel). And the LRP1 protein was normalized to that of GAPDH. The 0 h time point was arbitrarily set to 100% (lower panel). (**j**)Time-dependent Caspase 3/7 activities after CHX (50 μg/ml) of LRP1 and YAP proteins in the indicated MUM-2B cells. The blue line was represented the control group; The red line was represented the YAP-FLAG group. (**k**) Time-dependent Caspase 3/7 activities after CHX (50 μg/ml) of YAP in the indicated MUM-2B cells. The blue line was represented the control group; The red line was represented the YAP-FLAG group. (**l**) Relative mRNA of LRP1 in melanoma MUM-2B cells infected with GFP-sh or YAP-sh1 or YAP-sh2. (**m**,**n**) Immunofluorescence assays using anti-YAP and anti-LRP1 antibodies (lower panel). Scale bar, 10 μM. (**o**,**p**) Knockdown of YAP inhibited the activity of LRP1 promoter and over-expressed YAP stimulated the activity of LRP1 promoter, LRP1 promoter containing −2000 bp to +150 bp luciferase activities were measured in melanoma MUM-2B cells with control which were transfecting PGL4/Basic. Data were shown as mean ± SD from three independent experiments (including WB). *P < 0.05; **P < 0.01; ***P < 0.001 versus control measured by the student *t* test.

Since the knockdown of YAP resulted in significant down-regulation of LRP1 (Figs 4g,h and 5g,h), we were interested in investigating how YAP induces the expression of LRP1. We found that the degradation of LRP1 induced by the protein synthesis inhibitor cycloheximide (CHX) could be prolonged by overexpression of YAP (Figs 4i–k and 5i–k). Therefore, we tested if YAP affected LRP1 at the transcription level. Next, we found that the knockdown of YAP resulted in decreased LRP1 mRNA levels (Figs 4l and 5l). To investigate whether LRP1 is co-localized with YAP in melanoma A375 cells and MUM-2B cells, we performed IF analysis with anti-YAP and anti-LRP1 antibodies and found that YAP was not co-localized with LRP1 (Figs 4m,n and 5m,n). LRP1 was localized mostly in the nucleus, and YAP was localized in both the nucleus and cytoplasm. Then, we constructed an LRP1 promoter luciferase reporter system to confirm whether YAP regulates LRP1 activity at the transcription level. We discovered that luciferase activity of the LRP1 promoter was largely enhanced by transfecting the YAP-FLAG plasmid into melanoma A375 cells and MUM-2B cells. Activity of the LRP1 promoter was inhibited by transfecting the YAP-sh plasmid into melanoma A375 cells and MUM-2B cells, when compared to those infected by the GFP-sh plasmid (Figs 4o,p and 5o,p). Therefore, we have concluded that YAP affects the expression of LRP1 mainly through influencing the transcription of LPR1with affecting protein stability.

### LRP1 reversed impaired pro-carcinogenic function in melanoma cells with YAP-KD

**Figure 6:**
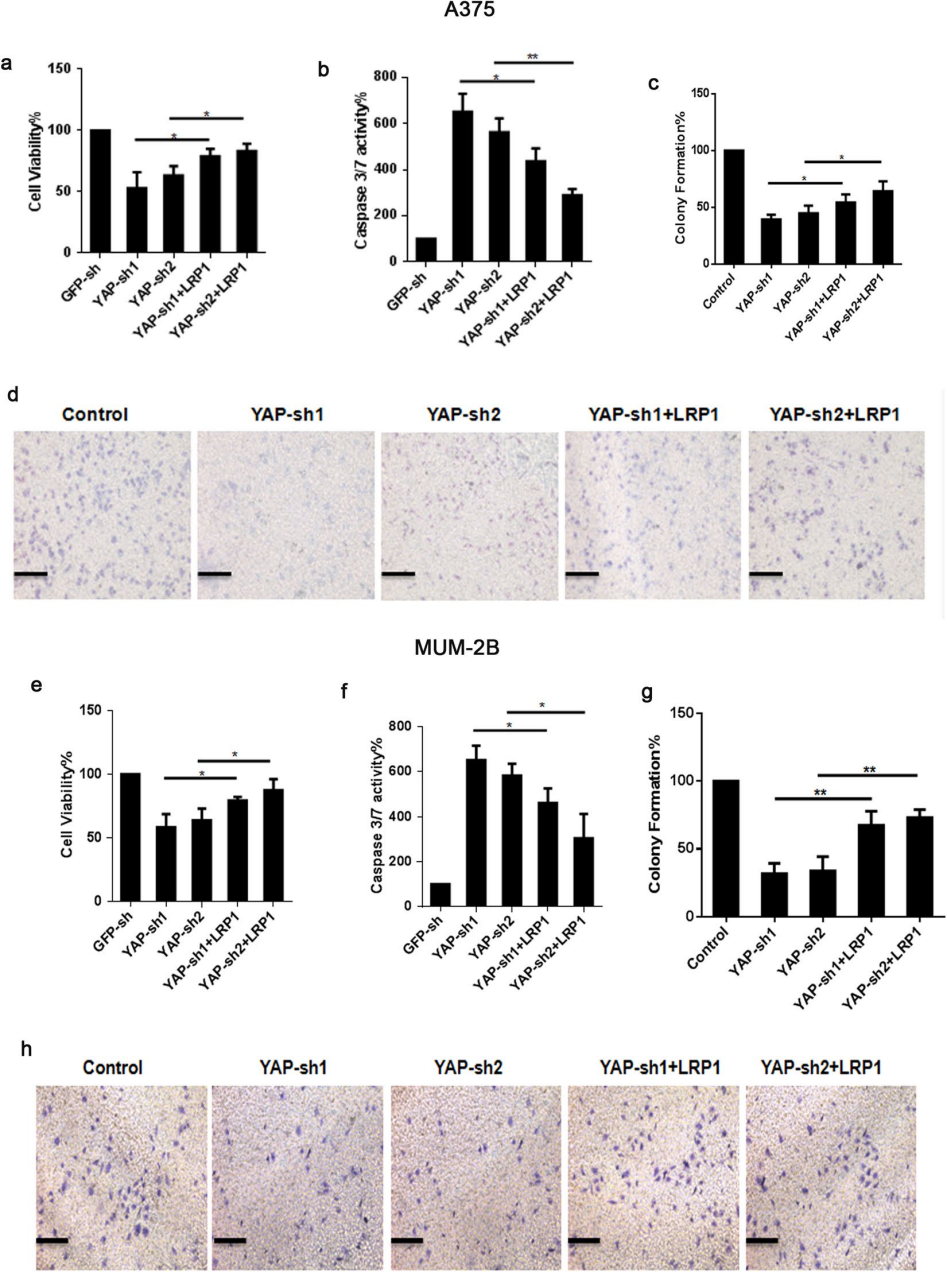
LRP1 reversed impaired pro-carcinogenic function in melanoma cell with YAP-KD. (**a**) Cell proliferation in melanoma A375 cells was measured by MTT assays when transfected with different expressing plasmids as indicated. (**b**) Caspase 3/7 activities in melanoma A375 cells were measured when transfected with different expressing plasmids as indicated. (**c**,**d**) Transwell activity in melanoma A375 cells was measured by transwell assay when transfected with different expressing plasmids as indicated. (**e**) Cell proliferation in melanoma MUM-2B cells was measured by MTT assays when transfected with different expressing plasmids as indicated. (**f**) Caspase 3/7 activities in melanoma MUM-2B cells were measured when transfected with different expressing plasmids as indicated. (**g**,**h**) Transwell activity in melanoma MUM-2B cells was measured by transwell assays when transfected with different expressing plasmids as indicated. Data were shown as mean ± SD from three independent experiments. *P < 0.05; **P < 0.01 versus control measured by the student *t* test.

**Figure 7:**
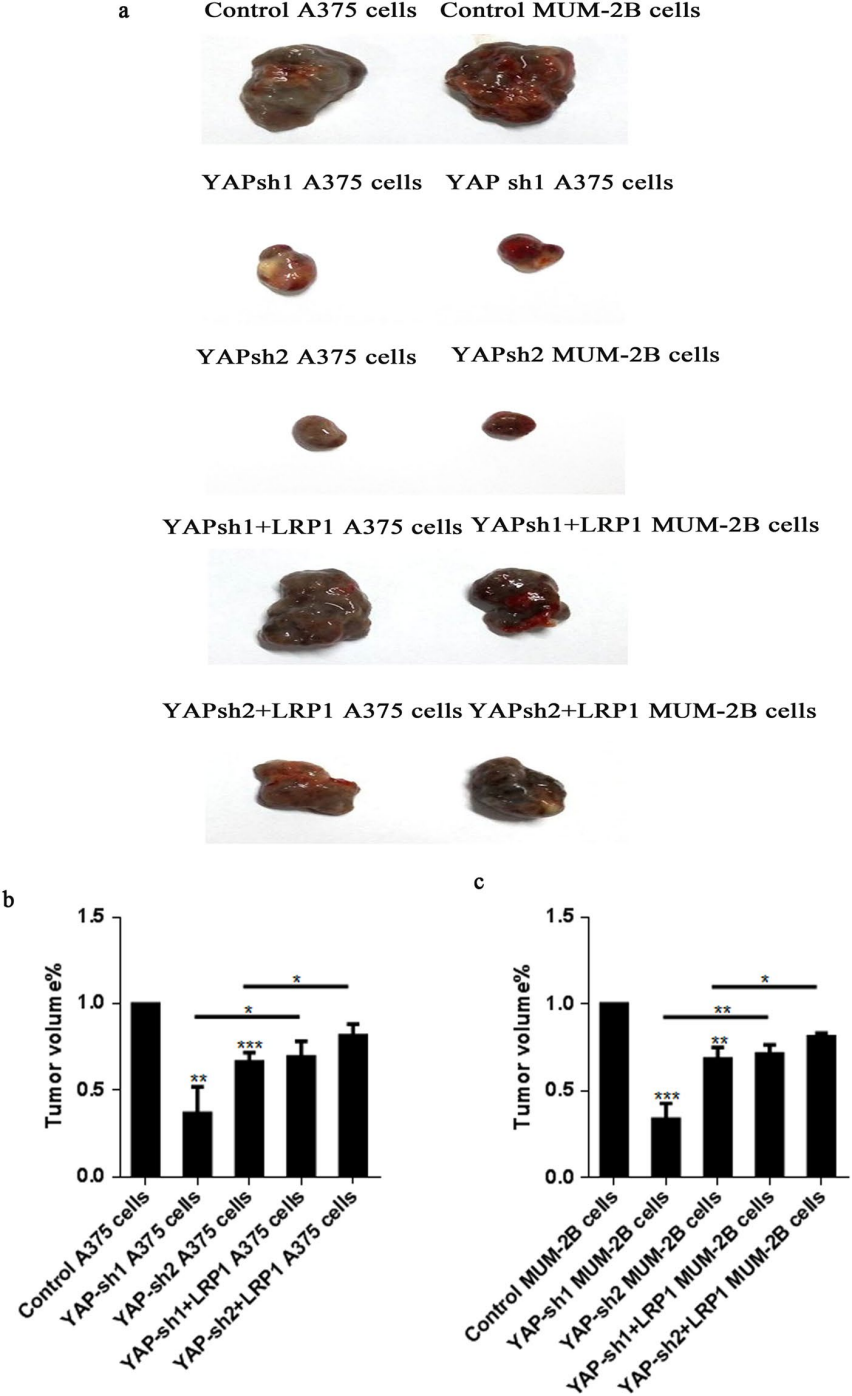
LRP1 reversed impaired pro-carcinogenic function in *vivo* with YAP-KD. (**a**) A375 melanoma cells and MUM-2B melanoma cells (2 × 10^5^) were transfected with GFP-sh or YAP-sh or YAP-sh + LRP1-FLAG and then injected subcutaneously to athymic nude mice (n = 5 for each group). Three weeks later, cohorts of mice from each group were sacrificed to determine the tumor volume, the *in vivo* experiment was repeated *twice*. (**b**) Representation (up) and quantification (down) of xenograft tumors formed by subcutaneous injection of A375 melanoma cells stably transfected with YAP-sh plasmids or infected with YAP-sh + LRP1-FLAG plasmids. (**c**) Representation (up) and quantification (down) of xenograft tumors formed by subcutaneous injection of MUM-2B melanoma cells stably transfected with YAP-sh plasmids or infected with YAP-sh + LRP1 plasmids. *P < 0.05; **P < 0.01; ***P < 0.001 versus control measured by the student *t* test.

We previously showed that YAP and LRP1 played their carcinogenic roles by inhibiting apoptosis and enhancing cell viability. Here, we further investigated whether LRP1 was important for the anti-apoptotic role of YAP in melanoma A375 cells and MUM-2B cells. We found that knocking YAP down led to a significantly increased caspase-3/7 activity, and such an effect could be partially reversed by simultaneous overexpression of LRP1 in A375 cells (Fig. 6b) and MUM-2B cells (Fig. 6f). Moreover, we revealed that the reduced cell proliferation and colony formation caused by knocking down YAP could also be partially reversed by simultaneous overexpression of LRP1, suggesting that the pro-tumorigenic function of YAP may rely on LRP1 (Fig. 6a,c,d,e,g,h). Therefore, we concluded that the carcinogenic function of YAP may rely on LRP1 in melanoma cells (Fig. 7).

**Figure 8:**
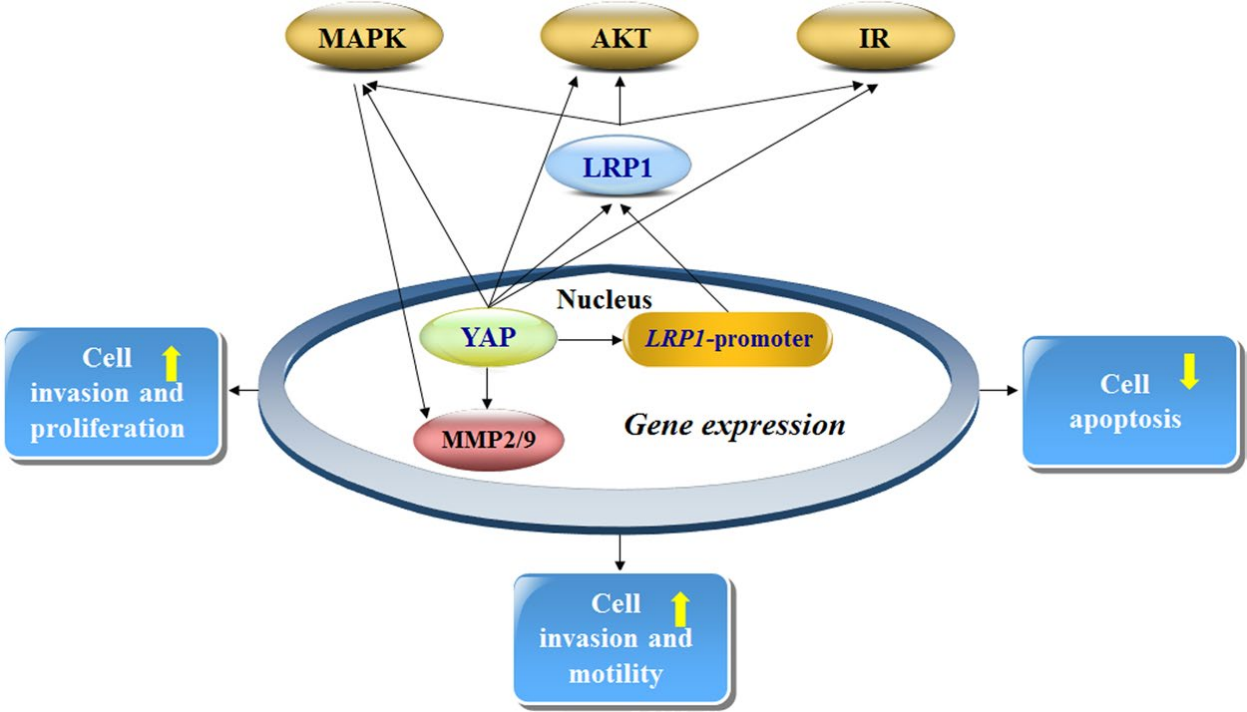
Possible mechanisms underlying how YAP regulated LRP1 by promoting proliferation and metastasis of melanoma cells and also reducing apoptosis. These pathways are involved in several processes of tumorigenesis and progression. Yes-associated Protein (YAP) can stimulate the expression of Low-density lipoprotein receptor-related protein 1(LRP1) via binding to the LRP1-promoter.LRP1 can activates the mitogen-activated protein kinase signaling pathway (MAPK), which can increase the transcriptional levels of matrix metalloproteinase (MMP)2 and MMP9. MMP2/9 can drive cancer cell proliferation and invasion.LRP1 activates the serine/threonine protein kinase (AKT) signaling pathway and insulin receptor (IR), which inhibits cancer cell apoptosis.

### LRP1 reversed impaired pro-carcinogenic function *in vivo* with YAP-KD

To further investigate whether knockdown of YAP or LRP1 could inhibit melanoma growth *in vivo*, A375 and MUM-2B cells were transfected with GFP-sh/YAP-sh/YAP-sh + LRP1-FLAG to produce subcutaneous tumors in athymic nude mice (Fig. 8a). Based on the close relationship between YAP and LRP1 *in vitro* and the TMA, we investigated the growth of A375 cell or MUM-2B cell clones after injection into the athymic mice. Compared to the control, we found that knockdown of YAP led to a dramatic inhibition of the xenograft growth. Interestingly, the reduction of YAP-induced inhibition of tumor growth could be gradually rescued by overexpression of the LRP1 plasmid, demonstrating that LRP1 is an important downstream effector of YAP *in vivo* (Fig. 8b,c).

## Discussion

Tumorigenesis is a synergistic procedure that includes cell transformation, invasive growth, angiogenesis and spread of the tumor into distant sites^10^. Malignant melanoma is one of the deadliest and most treatment-resistant cancers, with an increasing incidence rate worldwide. Despite the fact that great breakthroughs in targeted therapies have revolutionized the treatment strategies for metastatic melanoma, durable clinical responses remain elusive. The Hippo signaling pathway regulates tissue regeneration, organ size and stem cell self-renewal, and YAP is the key downstream transcription co-activator in this pathway. It has been shown that YAP is involved in the regulation of tumorigenesis. Flore Nallet-Staub *et al*.^11^ observed that YAP contributes to the invasive and metastatic capacity of melanoma cells and may represent a worthy target for therapeutic intervention. YAP is expressed in the nucleus and cytoplasm in the epidermis^12^.

LRP1, also known as CD91, is a multifunctional endocytic and cell signaling receptor. LRP1 is widely expressed on the surface of multiple cell types, such as neurons, astrocytes, smooth muscle cells and malignant cells. LRP1 is critically involved in tumor cell invasion and tumor progression. LRP1 not only regulates tumor invasion and migration through metalloproteinase MMP-2 and MMP-9 expression, but it also inhibits cell apoptosis by affecting caspase-3^13^. However, LRP1 is a controversial protein in the pathogenesis of tumor development. Li and colleagues^14^ have found that LRP1 promotes invasiveness of breast cancer cells *in vitro*. In contrast, previous studies have shown that LRP1 inhibits development and aggressiveness in several human tumor cell lines, including lung carcinoma and osteosarcoma, compared with non-tumor cell lines^15,16^. Therefore, the function of LRP1 in cell migration and invasion likely depends on the tumor cell type and the specific extracellular proteins involved in these processes. In prostate cancer, LRP1 expression is observed mostly in tumors with a high Gleason grade, which are the most progressive tumors; however, in hepatocellular carcinoma, a reduction in LRP1 expression may correlate with tumor progression. In this study, we have revealed a positive relationship between the expression of the two proteins YAP and LRP1, which can promote the transformative phenotypes of melanoma A375 cells and MUM-2B cells *in vitro* and *in vivo*. Mechanically, overexpression of YAP leads to increased expression of LRP1, and lowered expression of YAP leads to decreased expression of LRP1. YAP can positively regulate LRP1 by influencing the activity of the *LRP1* promoter.

It has been reported that LRP1 plays an important role in endocytosis and the regulation of most signaling pathways. LRP1 is also implicated in several physiologic processes, including regulation of the proliferation of vascular smooth muscle cells, lipid metabolism, and neuro-development^17^. From a mechanistic standpoint, LRP1 demonstrates substantial activity that may regulate cancer cell physiology *in vitro* and in preclinical mouse model systems^18^. LRP1 regulates uPAR signaling via factors such as ERK-1/2, which promotes tumor cell survival, proliferation, migration and invasion. The RAS/RAF/MEK/ERK pathway, which includes ERK-1/2, has been reported to be activated in over 80% of all cutaneous melanomas^19^. In the pathogenesis of melanoma tumorigenesis, LRP1 may be a crucial protein. Therefore, we performed an MTT-based assay and a transwell assay and measured caspase-3/7 activity to investigate if LRP1 had an effect on the transformative phenotypes of melanoma cells. Ultimately, we identified that knockdown of the LRP1 protein could inhibit proliferation and metastasis of melanoma cells, the same effect produced by transfecting the YAP-sh plasmid into melanoma cells. Our data are consistent with our hypothesis and provide important information that YAP and LRP1 have similar functions in maintaining the transformative phenotypes of melanoma A375 cells and MUM-2B cells. We also transfected YAP-FLAG and LRP1-FLAG into melanoma A375 cells and MUM-2B cells, and the results showed that both promoted transformative phenotypes of A375 cells and MUM-2B cells. It was further indicated that the expression of YAP was closely correlated with the expression of LRP1 in melanoma skin tissues. However, how YAP regulates LRP1 in the A375 cells and MUM-2B cells remains poorly understood. We revealed that CHX-induced protein degradation of LRP1 could not be prolonged by overexpression of YAP. In addition, YAP regulated LRP1 by affecting the transcription of LRP1. YAP also stimulated the activity of the *LRP1* promoter^20–22^. A study has revealed that YAP can be a potential therapeutic strategy in uveal melanoma with mutated GNAQ and GNA11^23^. YAP is commonly activated in many *in vitro* and *in vivo* models of tumorigenesis, as well as a number of human cancers, and provides an attractive target for potential therapeutic intervention. However, YAP is usually located in the cell nucleus, and consequently it is difficult to test and give a targeted therapy. In this experiment, we have found a relationship between YAP and LRP1 in the tumorigenesis of melanoma. LRP1 is widely located on the surface of multiple cell types, and it is easy to test. Therefore, LRP1 may be a potentially feasible protein to therapeutically target in the treatment of melanoma with mutated YAP. This study is also the first experiment to reveal the role of LRP1 in the mechanism of melanoma tumorigenesis and to explore the relationship between YAP and LRP1.

However, there were several limitations in the elucidation of the nature of the relationship between YAP and LRP1. First, LRP1 was not knocked down to determine if LRP1 has a feedback effect on the regulation of YAP. Second, we did not confirm the YAP binding site within the LRP1 promoter. Third, due to the limited number of melanoma patients in China, we did not validate the relationship between YAP and LRP1 proteins in melanoma samples that included blood and cutaneous tissues.

In summary, our results provided comprehensive evidence that YAP and LRP1 levels were increased in both A375 and MUM-2B cells. Furthermore, YAP stimulated expression and activity of LRP1 through promoter binding *in vitro* and reversed impaired pro-carcinogenic function *in vitro* and *in vivo* with YAP-KD. Thus, it could advance the field and provide the basis for further study of the LRP1 inhibitor in cutaneous melanoma.

## Methods

### Cell culture, transfection and lentiviral infection

The A375 human melanoma cell lines were provided by the Cell Bank of the Chinese Academy of Sciences. The MUM-2B human melanoma cell lines were provided by the JI Kai Gene Company. A375 and MUM-2B cells were cultured in DMEM supplemented with 10% fetal bovine serum (FBS, Gibco, Grand Island, NY, USA). Cells were treated with cycloheximide (CHX, 50 µg/ml, Sigma, St. Louis, MO, USA) for the time indicated in the CHX chase experiments. For lentiviral infection, lentiviral-based constructs were transfected along with the packaging plasmids into growing HEK293T cells. Viral supernatants were collected 48 hours after transfection. Target cells were infected in the presence of polybrene and then subjected to selection with puromycin.

### Cell proliferation, caspase-3/7 activity, transwell assay and quantitative RT-PCR (qPCR)

Cell proliferation was measured with a CellTiter Proliferation Assay kit (Promega), and caspase-3/7 activity was determined using a Caspase-Glo Assay kit (Promega), in accordance with the manufacturer’s instructions.

Cell invasion assays were performed using 24-well transwells (8 µm pore size; Corning Incorporated, Corning, NY, USA) pre-coated with Matrigel (BD Biosciences, #Falcon354480). Cells on the lower surface of the membrane were fixed in 4% paraformaldehyde and stained with crystal violet. Cells in 5 microscopic fields (100x magnification) were counted and photographed under a Leica fluorescence microscope (DMI6000B). All experiments were performed in triplicate.

The mRNA levels were measured by qPCR, and experiments were performed as previously described^24^. GAPDH was treated as an internal control. Primers were used as follows: GAPDH, sense: 5′-ATCATCCCTGCCTCTACTGG-3′, anti-sense: 5′-GTCAGGTCCACCACTGACAC-3′; YAP, sense: 5′-CCTCGTTTTGCCATGAACCAG-3′, anti-sense 5′-GTTCTTGCTGTTTCAGCCGCAG-3′; LRP1, sense: 5′-ACCTACAAGATGTACGAAGGC-3′, anti-sense: 5′-ATGTAGAGTGTGGCATACACG -3′. Each sample was tested in triplicate.

### Western blotting (WB), immunofluorescence (IF) and immunohistochemistry (IHC)

For WB, proteins were resolved on SDS-polyacrylamide gels followed by a standard WB. The primary antibodies included anti-YAP (CST, Boston, MA, USA, #4912), anti-LRP1 (Epitomics, Burlingame, California, MA, USA, #2703) and anti-GAPDH (CST, #5714).

For IF, cells were fixed with 4% paraformaldehyde (PFA) for 15 min, washed with PBS and blocking buffer (3% FBS + 1% heat-inactivated sheep serum + 0.1% Triton X-100), and then incubated overnight at 4 °C in primary antibodies against YAP (CST, #4912), LRP1 (Epitomics, #2703), cleaved caspase-3 (Cell Signaling Technology (CST) Boston, MA, USA, #9664). Alexa-Fluor-488 or 555 fluorescent conjugated secondary antibodies (Invitrogen, Carlsbad, CA, USA) were used for detection.

For IHC, melanoma tissue microarray analysis (TMA) slides were purchased from Biomax (Rockville, MD, USA). Sections were incubated overnight in primary antibodies against YAP or LRP1. Each tissue sample was scored using a semiquantitative scale on the array slide, with + for weak staining (i.e., 20–40% of the cells showed weak to intermediate intensity staining), ++ for strong staining (i.e., ≥10% of the cells showed very intense staining or 50% of the cells showed weak to intermediate intensity staining, in an appropriate subcellular distribution), +++ for very strong staining (i.e., ≥30% of the cells showed very intense staining or >80% of the cells showed moderately intense staining).

### Luciferase reporter assay

The backbone plasmid of the luciferase reporter was pGL4.21, which was purchased from Promega (Madison, WI, USA). The promoter region (−2000 bp to +150 bp) of the *LRP1* gene was PCR amplified using primers (sense: ATCGGGTACCACATTCACATTCAGGAGACAGCAGG; anti-sense: ATCGGATATCAAATGCACAATTGGGAGGAGGCGGG) and cloned into pGL4.21 plasmids. A375 cells and MUM-2B cells were co-transfected with luciferase reporter plasmids and pRL-TK Renilla reporters with or without overexpression/knockdown of YAP. The YAP-FLAG and YAP-sh plasmids were gifts from the Jiayi Wang lab (Shanghai Tenth People’s Hospital, Tongji University School of Medicine, Shanghai, China). Luciferase activity was measured by the Dual-Luciferase Reporter kit (Promega, Madison, WI, USA) after transfection.

### Xenograft mouse experiments

Control and Yap-knockdown or YAP-knockdown + LRP1 A375 cells (2*10^5^ cells) (infected with YAP-sh1/YAP-sh2 or lentiviral LRP1-myc expressing plasmids, respectively) were subcutaneously injected into the flanks of athymic nude mice (6-week-old, Bikai, Shanghai, China). The tumor size was measured. Tumor length (*L*) and width (*W*) were measured at the end of the experiment. Tumor volume was estimated by the formula 0.5 * (L * W^2^).

### Statistical Analysis

Data are presented as the mean ± SD. Statistical analysis was performed with Microsoft Excel 2007 (Microsoft Corporation, NY, USA) and GraphPad Prism software (GraphPad, San Diego, CA, USA) using a two-tailed Student’s *t* test and *X* ^2^ test. *P < 0.05 was regarded as statistically significant.
